# Enhancing Time-Domain Interference Alignment for Underwater Acoustic Networks with Cross-Layer Design

**DOI:** 10.3390/s25010068

**Published:** 2024-12-26

**Authors:** Qiao Xiao, Zhicheng Bi, Chaofeng Wang

**Affiliations:** 1 School of Computer Science, University of South China, Hengyang 421001, China; qiaox@usc.edu.cn; 2 School of Electrical Engineering, University of South China, Hengyang 421001, China; zcbi@stu.usc.edu.cn

**Keywords:** underwateracoustic networks, transmission scheduling, power allocation, time-domain interference alignment, cross-layer design

## Abstract

In exploiting large propagation delays in underwater acoustic (UWA) networks, the time-domain interference alignment (TDIA) mechanism aligns interference signals through delay-aware slot scheduling, creating additional idle time for improved transmission at the medium access control (MAC) layer. However, perfect alignment remains challenging due to arbitrary delays. This study enhances TDIA by incorporating power allocation into its transmission scheduling framework across the physical and MAC layers, following the cross-layer design principle. The proposed quasi-interference alignment (QIA) mechanism enables controlled interference on useful signals by jointly optimizing the transmission schedule and power. The formulated optimization problem to maximize network throughput is divided into two sub-problems: one for coarse slot scheduling and another for refining both scheduling and power allocation. The simulation results validate the QIA framework’s superiority over the traditional TDIA and genetic algorithm benchmarks.

## 1. Introduction

Acoustic signals travel in water with lower attenuation compared to optical or electromagnetic ones, enabling relatively reliable underwater communications in the medium and long ranges [1,2,3]. Underwater acoustic (UWA) communication networks have gained significant attention in recent years as they are suitable for facilitating underwater information exchange in various ocean-related applications, such as oil drilling, environmental monitoring, and disaster early warning, which have become increasingly vital for human development and safety [4,5].

Compared to terrestrial radio networks, UWA networks encounter diverse challenges [6] such as high channel dynamics, low bandwidth, large propagation delays, and limited power supply. Among the challenges, the significant propagation delay, an inherent characteristic of underwater acoustic (UWA) communication due to the relatively slow speed of sound in water, approximately 1500 m/s, presents considerable difficulties in designing effective transmission protocols for UWA networks [7]. Due to the large propagation delay, UWA networks suffer from spatial–temporal (ST) uncertainty [8,9,10], where transmission interference and collisions arise from not only the transmission schedule but also the spatial locations of network nodes. This uncertainty would make the interference relationship between UWA transmission links highly complicated, rendering protocols designed for terrestrial radio networks inapplicable in UWA networks.

In the literature, many existing studies have treated the large propagation delays in UWA networks as detrimental, given their spatial–temporal uncertainties that negatively impact transmission performance [11,12,13]. Hence, most of them ultimately regard the propagation delays as detrimental and focus on delay-tolerant MAC designs to enable concurrent transmissions in the presence of the delays. However, some works [14,15,16] take advantage of the large propagation delays to improve the performance of UWA networks based on time-domain interference alignment (TDIA). Specifically, the interference signals are aligned exactly together in the time domain to reserve more available time for transmitting useful signals. By optimizing the transmission schedule from the MAC layer perspective, these methods could achieve significant throughput improvements in UWA networks. It is worth noting that most of these works assume a time-slotted transmission structure, where each transmission of a signal block occupies one time slot. In this case, exploiting TDIA to maximize the throughput by determining whether to transmit in a slot can be formulated as combinatorial optimization problems [17,18]. However, solving such problems can be computationally intensive and require specialized algorithms to find optimal solutions, which may become stuck in local optimal. Moreover, the propagation delay is typically considered as an integer multiple of the duration of one time slot to simplify the system models. This can be performed by either rounding off the propagation delay straightforwardly or adapting the length of signal blocks, even the duration of a time slot, to match the propagation delay [14]. In practice, these approaches could either violate the principle of collision-free transmission for useful signals under the TDIA mechanism or introduce another layer of complexity in system design.

In this work, inspired by TDIA performed at the MAC layer, we propose a cross-layer design-based transmission mechanism, i.e., quasi-interference alignment (QIA), to jointly optimize the power allocation and transmission schedule. The fundamental concept of QIA is to expand the potential time slots available for data transmission by closely aligning the received interference in time while allowing for minor overlaps between interference and useful signals. This approach aims to enhance transmission capacity by jointly optimizing the power allocation and transmission schedule, from the perspectives of the MAC and physical layers, to reduce overall reception time while collaboratively mitigating interference arising from these slight collisions, respectively.

### 1.1. A Motivational Example for QIA

**Figure 1:**
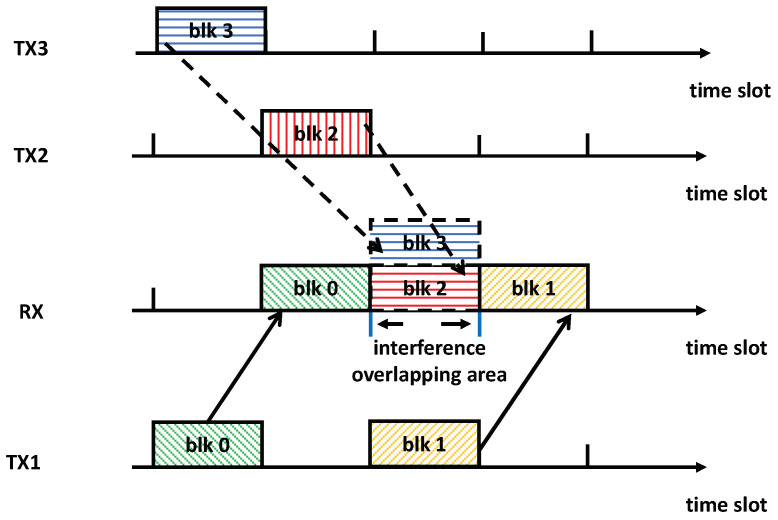
Ideal time-domain interference alignment.

The ideal receiving pattern in the time domain of incoming signal blocks at a receiver with TDIA can be depicted in Figure 1, assuming that the propagation delays between transmitter–receiver pairs are measured in integer multiples of a time slot. Specifically, the transmitter TX1 communicates with the receiver RX, while the transmitters TX2 and TX3 communicate with their respective destinations rather than RX. Hence, TX2 and TX3 would interfere with the transmission link between TX1 and RX. The propagation delays from TX2 and TX3 to RX are 1 and 2 time slots, respectively. One can see that the interference signals at RX are overlapped perfectly within a single time slot based on TDIA. Moreover, the received useful signals are collision-free from the interferences, in accordance with the MAC rules. Hence, it represents an ideal scenario viewed from the MAC layer where the reception time for a receiver is fully utilized and collisions are entirely avoided.

**Figure 2:**
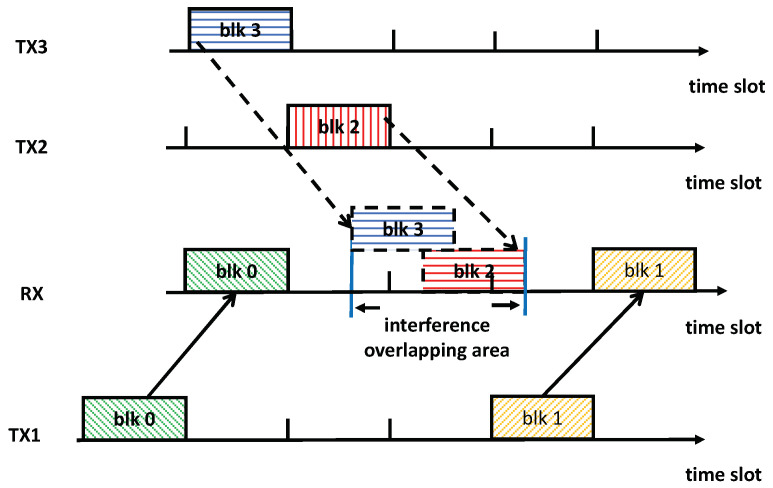
TDIA with arbitrary propagation delays.

However, due to arbitrary propagation delays, the perfect TDIA pattern does not hold for time-slotted systems in practice. For instance, consider the scenario depicted in Figure 2, the propagation delays from TX2 and TX3 to RX are now 1.2 and 1.8 time slots, respectively. In this situation, if the system adheres to the original schedule established by TDIA in Figure 1, the interference blocks 2 and 3 will no longer be aligned perfectly, while collisions occur between blocks 0 and 3, as well as between blocks 1 and 2. As illustrated in Figure 2, if the transmission schedule of blocks 0 and 1 is adjusted to avoid collisions, it would result in a significant amount of time being wasted at RX during the reception, potentially leading to a substantial loss in network throughput.

It can be seen that without the time-slotted transmission structure, the perfect TDIA could potentially be achieved for the example in Figure 2. However, please note that, besides the transmission link of TX1 to RX, there exist numerous other concurrent transmission links. For these links, with their arbitrary propagation delays and TDIA strategy performed, there is a great chance that the interference signals received at all the receivers will not perfectly overlap in the time domain. As a result, the possibility of reception time being wasted across the whole system due to non-overlapping interference signals would be considerably high. Hence, not utilizing the time-slotted transmission structure will only contribute to saving some of the waste in the total reception time but cannot completely eliminate it.

**Figure 3:**
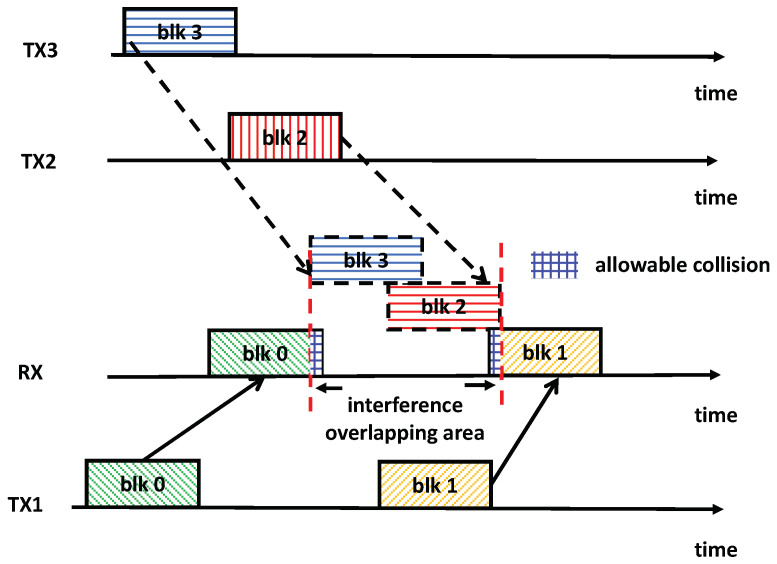
The proposed quasi-interference alignment.

In reality, the proposed QIA mechanism essentially aims to mitigate the interference following the principle of TDIA. It could help to relax the constraint imposed by the traditional TDIA, wherein the received useful signals must not overlap with interference signals, providing additional degrees of freedom (d.o.f.) to enhance network throughput. As illustrated in Figure 3, even though blocks 0 and 1 are affected by the interference blocks 3 and 2, respectively, the total reception time can be heavily reduced compared to the situation presented in Figure 2. Moreover, the transmission power of TXs 2 and 3 can be adjusted to ensure that their transmitted blocks can be decoded by the corresponding receivers while the useful signal blocks 1 and 0, even when colliding with interference signals, can still be decoded by RX. In this way, the transmission power and schedule can be jointly optimized to maximize the network throughput.

### 1.2. Our Contributions

The traditional TDIA is performed at the MAC layer, which optimizes the transmission schedule to overlap interference and strictly avoids collisions on useful signals, thereby enhancing the network throughput. Nevertheless, from the perspective of the physical layer, it is still possible to decode information even in the presence of minor collisions as long as the useful signals have satisfactory received signal-to-noise and interference ratio (SINR) [19]. In this work, taking advantage of the cross-layer design, the strict restriction imposed by TDIA on the transmission schedule is relaxed while power allocation is introduced to compensate for any negative effects arising from this relaxation of the constraint. A throughput optimization approach is proposed that integrates power allocation at the physical layer with transmission scheduling in the original TDIA framework at the MAC layer. However, the integration of the cross-layer design introduces a higher level of computational complexity, particularly when dealing with continuous optimization variables for both transmission power and transmission starting time. Hence, to address the elevated complexity, efficient algorithms are proposed to obtain the optimal solution for the optimization problem established based on QIA.

The main contributions of our work are as follows:

- We design a TDIA-based mechanism, termed QIA, which leverages a cross-layer design to jointly optimize transmission power and scheduling, aiming to improve overall network throughput. Beyond traditional scheduling, power allocation is incorporated to counteract the effects of permissible interference on useful signals, thereby providing additional d.o.f. for throughput enhancement.
- We reformulate the TDIA-based problem into a mixed integer linear programming (MILP) framework, enabling efficient solutions and laying the groundwork for addressing the QIA-based problem.
- We propose a two-step optimization strategy to effectively manage the increased computational complexity associated with the cross-layer design. It decomposes the throughput optimization problem into two sub-optimization problems, each of which can be efficiently solved with a smaller solution searching space.
- The proposed QIA algorithms are validated through simulations, showing higher system throughput compared to traditional TDIA, thereby demonstrating their efficacy.

This study considers enhancing the TDIA for UWA networks with the cross-layer design. Specifically, the proposed approach intends to maximize the utilization of TDIA patterns through strategic transmission scheduling, while simultaneously handling collisions on useful signals through effective power allocation. This approach could leverage a higher transmission d.o.f. compared to the traditional TDIA to achieve enhanced network throughput.

### 1.3. Paper Organization

The rest of the paper is organized as follows. The related literature is reviewed in Section 2. The system model of performing the proposed QIA is described in Section 3. The QIA-based algorithms which allow for the minor collision of useful signals to maximize the network throughput is proposed in Section 4. A performance evaluation of the proposed algorithms is presented in Section 5. Conclusions are drawn and future works are presented in Section 7.

## 2. Related Works

The concept of TDIA is initially introduced in terrestrial radio networks to align interference to subspaces orthogonal to that of the desired transmissions [20], thereby allocating more transmission resources for useful signals. For wireless networks with large propagation delays, ref. [21] reveals that perfect TDIA can be achieved based on the assumption that the transmitted symbol duration can be arbitrarily small. For time division multiple access (TDMA)-based UWA networks, ref. [14] proposes a transmission scheduling method based on the TDIA mechanism, leveraging large propagation delays to optimize network throughput. Specifically, the transmission start time of signal blocks is adjusted to align interference signals perfectly within the same time slots, ensuring that useful signals are received in interference-free slots. Simulation results demonstrate that this approach achieves higher throughput compared to zero-delay communication systems such as terrestrial radio networks. Similar ideas have been applied to different UWA networks with different network types such as mulitcast UWA networks [15] and multi-hop UWA networks [22], as well as to different applications such as practical message scheduling [16]. However, since the propagation delays of transmission links can take arbitrary values, achieving perfect alignment of interference signals poses significant challenges. Some studies, such as refs. [14,16], assume that the length of data blocks can be freely adjusted to match the propagation delays. Others, like [15], approximate delays as integer multiples of time slots, ensuring that the received signals fully occupy a single time slot. To address arbitrary propagation delays, ref. [22] proposes a TDMA-based frame structure where time slots among different receivers are asynchronous, allowing received interference to span across two time slots. However, under the MAC mechanism, a time slot becomes invalid if any portion is occupied by interference, leading to inefficient utilization of time resources.

Furthermore, transmission scheduling in each time stamp can in fact be regarded as a sequential decision-making problem, which motivates researchers to explore reinforcement learning (RL), which could dynamically learn the optimal transmission schedule online for UWA networks [23,24,25]. Under the RL paradigm, the transmission actions are learned by accumulating rewards resulting in throughput improvement. In refs. [26,27], RL-based transmission scheduling algorithms for UWA networks are proposed to determine whether a node should transmit a message or remain idle for each time slot. The system receives positive rewards only when transmissions from individual node do not interfere with receptions in other links. Consequently, the TDIA principle can be indirectly promoted by allowing the transmitters to learn that adhering to TDIA would yield higher rewards.

It can be seen that most existing works consider TDIA as the MAC layer design problem and also the traditional time-slotted transmission with propagation delays measured in an integer number of time slots. Those works either formulate TDIA as a constrained optimization problem to enforce interference-free transmissions while design efficient solvers seek for the optimal transmission schedule or exploit online learning to learn the optimal transmission schedule, which could result in TDIA patterns.

In recent years, cross-layer design approaches have gained significant attention for improving network throughput, particularly through the integration of joint transmission scheduling and power allocation [28,29]. In these studies, the transmission process of a signal block is organized in the time-slotted style. However, due to the arbitrary propagation delays considered, the received blocks can span two time slots, leading to possible collisions on useful signals. Yet, these studies consider that this situation could offer the opportunity to optimize the network throughput by allocating transmission power to ensure that even the interfered useful signals can be decoded with satisfied SINRs. As the transmission schedule in the two studies is treated as a discrete optimization variable, a mixed integer optimization problem can be established and efficient solvers are proposed accordingly.

In this work, following the line of TDIA, we adopt a strategy of integrating power allocation in TDIA to enhance the network throughput by considering the arbitrary propagation delays. The strategy intends to enforce the TDIA constraint as effectively as possible, while also ensuring that the received useful signals can be decoded successfully. This approach could strike a balance between TDIA and signal reception quality, ultimately contributing to an overall performance improvement in network throughput.

## 3. System Model

**Figure 4:**
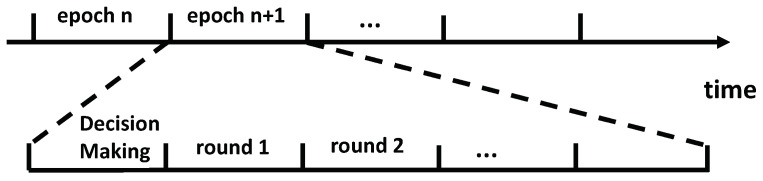
Transmission structure of epochs.

In this work, as shown in Figure 4, we consider that UWA networks operated in an epoch-based system and one epoch can be further divided into multiple rounds. To mitigate time wastage caused by arbitrary propagation delays in the time-slotted system, as illustrated in Figure 2, each round allows the transmission of a signal block to begin at any point within the round. This approach optimizes the likelihood of interference alignment patterns, thereby enhancing the achievable network throughput. It is assumed that the transmitters and their designated receivers have already been pre-selected to meet specific application requirements, such as multi-hop routing or time-sensitive scheduling. We assume that there are *N* active transmission links, where the *m*th transmitter is paired with the *m*th receiver, without loss of generality. That is, for m≠n, the signal transmitted by the *m*th transmitter will interfere with the signal reception at the *n*th receiver. These active links maintain their transmission strategy throughout the entire epoch. Within each round of an epoch, all the active transmitters intend to transmit *L* acoustic signal blocks to their destination receivers. All the signal blocks have the identical signal duration of $T_{\mathrm{bl}}$. For underwater acoustic networks with a large number of nodes, similar to [29], we could divide the network into smaller clusters and implement the proposed method within each cluster to manage a smaller number of nodes effectively.

We assume a central scheduler in the network responsible for jointly determining the transmission parameters for all transmitters during a transmission epoch. In this work, the parameters include the transmission power and the transmission slot of each signal block to be transmitted. At the start of each epoch, the scheduler determines the optimal transmission parameters and disseminates them to the active transmitters. The central scheduler, typically a gateway or cluster head with enhanced computational capabilities, is able to efficiently manage the scheduling tasks. We further assume a perfect time synchronization across the entire network.

In the following, the time-of-arrivals (ToAs) and receiving patterns under the large propagation delays are presented and the mathematical formulation of the transmission capacity which serves as the optimization objective in this work is established.

### 3.1. Signal Reception with Large Propagation Delays

Let I(n) denote the set of transmitters whose transmissions interfere with the reception at the *n*th receiver. Specifically, for the *k*th transmitter, where k∈I(n), it transmits useful signals to the *k*th receiver but causes interference at the *n*th receiver. Denote $T_{m}^{\left(l\right)}$ as the transmission time of the *ℓ*th signal block from the *m*th transmitter, and let $\tau_{mn}$ represent the propagation delay between the *m*th transmitter and the *n*th receiver. Please note that, in the shallow water scenario, the delay $\tau_{mn}$ can be calculated based on the distance between transmission links and the speed of sound in water. In scenarios with stratification effects, the delay $\tau_{mn}$ can be determined based on the travel time of the signal along the strongest propagation path.

The time of arrival (ToA) of the *ℓ*th block from the transmitter *m* at the receiver *n* can be represented as

(1) $$t_{mn}^{\left(l\right)}=T_{m}^{\left(l\right)}+\tau_{mn}.$$

Consider the $l_{m}$th and $l_{k}$th blocks transmitted by the *m*th and *k*th transmitters, respectively. The ToA of these two blocks at the *n*th receiver can then be calculated as

(2) $$\begin{array}{cc} \; & t_{mn}^{\left(l_{m}\right)}=T_{m}^{\left(l_{m}\right)}+\tau_{mn}, \end{array}$$

(3) $$\begin{array}{cc} \; & t_{kn}^{\left(l_{k}\right)}=T_{k}^{\left(l_{k}\right)}+\tau_{kn}. \end{array}$$

Hence, the difference in ToAs between any two signal blocks at the receiver *n* can be expressed as

(4) $$\Delta_{mkn}^{\left(l_{m},l_{k}\right)}=t_{mn}^{\left(l_{m}\right)}-t_{kn}^{\left(l_{k}\right)}.$$

Thus, whether two signal blocks received by the *n*th receiver overlap with each other can now be indicated by the ToA difference $\Delta_{mkn}^{\left(l_{m},l_{k}\right)}$. Specifically, if the absolute value of the ToA difference is smaller than the duration of a time slot, i.e.,

(5) $$|\Delta_{mkn}^{\left(l_{m},l_{k}\right)}|<T_{\mathrm{bl}},$$

then the two signal blocks will overlap. On the other hand, if $|\Delta_{mkn}^{\left(l_{m},l_{k}\right)}|\geq T_{\mathrm{bl}}$, the $l_{m}$th and the $l_{k}$th two signal blocks sent by transmitters *m* and *k* are received without any collision. Based on this condition, the strategy of TDIA can be employed for transmission scheduling, ensuring that the received useful blocks are collision-free while maximizing the overlap of received interference signals, thereby optimizing network performance.

### 3.2. Transmission Capacity

In this work, we consider the transmission capacity as the optimization objective to maximize the network throughput. For the $l_{m}$th useful signal block transmitted by the *m*th transmitter and received by its designated destination—the *m*th receiver—according to ref. [30], its signal-to-interference-and-noise ratio (SINR) can be obtained by

(6) $$\lambda_{m}^{\left(l_{m}\right)}=\frac{P_{\mathrm{rx},mm}^{\left(l_{m}\right)}}{P_{0}+\frac{1}{T_{\mathrm{bl}}}\sum_{l_{m}=1}^{L}\sum_{k\in I(m)}P_{\mathrm{rx},km}^{\left(l_{k}\right)}{\left[T_{\mathrm{bl}}-\left|\Delta_{mkm}^{\left(l_{m},l_{k}\right)}\right|\right]}^{+}}$$

where *B* is the transmission bandwidth, $P_{0}\left(f_{c}\right)$ is the power spectral density of the ambient noise at the transmission center frequency $f_{c}$, and $P_{\mathrm{rx},mn}^{\left(l\right)}$ is the received power of the *ℓ*th signal transmitted by the *m*th transmitter to the *n*th receiver. According to ref. [31], the the ambient noise $P_{0}\left(f\right)$ can be obtained by

(7) $$P_{N}\left(f\right)=P_{t}\left(f\right)+P_{s}\left(f\right)+P_{w}\left(f\right)+P_{\mathrm{th}}\left(f\right),$$

where $P_{t}\left(f\right)$, $P_{s}\left(f\right)$, and $P_{w}\left(f\right)$ represent the noise contributions due to turbulence, shipping activity, surface motion and wind, respectively, and $P_{\mathrm{th}}\left(f\right)$ denotes the thermal noise. The detailed contributions of each noise source are described as follows:

(8) $$\begin{array}{cc} \; & 10\log P_{t}\left(f\right)=17-30\log f,\\ \; & 10\log P_{s}\left(f\right)=40-20\left(s-0.5\right)+26\log f-60\log\left(f+0.03\right)\log f,\\ \; & 10\log P_{w}\left(f\right)=50+7.5w^{0.5}+20\log f-40\log\left(f+0.4\right),\\ \; & 10\log P_{\mathrm{th}}\left(f\right)=-15+20\log f, \end{array}$$

where *s* is the shipping activity factor and *w* is the wind speed.

In addition, the term $[T_{\mathrm{bl}}-|\Delta_{mkm}^{\left(l_{m},l_{k}\right)}{\left|\right]}^{+}$ captures the effective collision duration, reflecting the overlap between the $l_{m}$th useful block and the $l_{k}$th interfering block transmitted by the *k*th transmitter. The operation ${\left[\cdot \right]}^{+}=:\max\left\{\cdot ,0\right\}$ ensures that only positive values of the collision duration are considered. In addition, the received power $P_{\mathrm{rx},mn}^{\left(l\right)}$ can be further given by

(9) $$P_{\mathrm{rx},mn}^{\left(l\right)}=P_{\mathrm{tx},m}^{\left(l\right)}\cdot G\left(f_{c},d_{mn}\right)$$

where $P_{\mathrm{tx},m}^{\left(l\right)}$ represents the transmission power of the *ℓ*th signal by the *m*th transmitter, and $G(f_{c},d_{mn})$ denotes the transmission gain as a function of the transmission center frequency $f_{c}$ in KHz and the propagation distance $d_{mn}$ in km, which can be characterized as

(10) $$G\left(f_{c},d_{mn}\right)=A_{0}{\left(d_{mn}\times 10^{3}\right)}^{-\beta }\alpha {\left(f_{c}\right)}^{-d_{mn}}$$

where $A_{0}$ is a normalization factor, β is the spreading factor, *l* is the link distance, $\alpha (f_{c})$ is the absorption coefficient, and $f_{c}$ is the transmission center frequency. According to [32], the coefficient α(f) at different frequencies can be obtained as

(11) $$10\log\alpha \left(f\right)=\frac{0.11f^{2}}{1+f^{2}}+\frac{44f^{2}}{4100+f^{2}}+2.75+10^{-4}f^{2}+0.003.$$

Based on the SINR of each received useful signal blocks, the transmission capacity at the *m*th receiver in a transmission round can be derived as

(12) $$C_{m}=\frac{T_{\mathrm{bl}}}{2}\sum_{l=1}^{L}\log_{2}\left(1+\lambda_{m}^{\left(l\right)}\right).$$

The total reception time at a specific receiver *m*, $T_{\mathrm{total}}^{\left(m\right)}$, can be obtained as the difference between the ToAs of the first and the last signal at the same receiver, plus the duration of a signal block, i.e.,

(13) $$T_{\mathrm{total}}^{\left(m\right)}=\max_{1\leq i,j\leq N;1\leq l_{i},l_{j}\leq L}\left\{t_{im}^{\left(l_{i}\right)}+T_{\mathrm{bl}}-t_{jm}^{\left(l_{j}\right)}\right\}.$$

Then, the total reception time of one round for the whole system, defined as the maximum reception time across different receivers to transmit all *L* blocks, can be described by

(14) $$T_{\mathrm{total}}=\max_{1\leq m\leq N}T_{\mathrm{total}}^{\left(m\right)}.$$

Finally, The total transmission capacity, summed over all receivers, is then given by

(15) $$C_{\mathrm{sum}}=\frac{1}{T_{\mathrm{total}}}\sum_{1\leq m\leq N}C_{m}.$$

## 4. Integrating Power Allocation in TDIA

According to (15), $T_{\mathrm{total}}$ is actually a function of the transmission starting time $T_{m}^{\left(l\right)}$ and the signal travel time $\tau_{mn}$ across all the active transmission links. In addition, the capacity at each receiver $C_{m}$ is influenced by the corresponding transmission power. Hence, to enhance the throughput network, optimizing the transmission schedule and power allocation is essential to maximize the total network capacity within a transmission round. The throughput maximization can therefore be expressed as the following optimization problem:

(16a) $$\begin{array}{cc} \{T_{m}^{\left(l\right)}, & P_{m}^{\left(l\right)}{\}}_{1\leq m\leq N,1\leq l\leq L}=\arg\max C_{\mathrm{sum}} \end{array}$$

(16b) $$\begin{array}{cc} s.t.\; & P_{\min}\leq P_{\mathrm{tx},m}^{\left(l\right)}\leq P_{\max}; \end{array}$$

(16c) $$\begin{array}{cc} \; & P_{\mathrm{rx},mm}^{\left(l\right)}\geq P_{\mathrm{th}},1\leq m\leq N; \end{array}$$

where $P_{\min}$ and $P_{\max}$ correspond to the minimum and maximum transmission power of the transmitters, respectively. The constraint (16c) ensures that the received SINR of useful signal blocks must exceed a threshold $P_{\mathrm{th}}$ to successfully decode the information.

It is important to note that (16) represents a comprehensive optimization problem aimed at determining both the transmission schedule and power allocation. However, due to the highly nonlinear nature of the operations in (16), it encompasses a vast feasible region, making the joint optimization of scheduling and power challenging. Traditional TDIA addresses this issue by focusing solely on transmission scheduling from the MAC layer perspective, excluding transmission power optimization under the assumption that interference-free signals can always be successfully decoded. As a result, the transmission schedule can be treated as a specific solution to (16).

In the following sections, we first formulate a throughput optimization problem that strictly adheres to the principles of traditional TDIA, optimizing only the transmission starting times under the MAC layer constraints. To facilitate solving the QIA-based problem, we reformulate the TDIA scheduling problem into an MILP framework that can be efficiently solved. Subsequently, we present the proposed approach, which permits slight overlaps with useful signals and integrates power allocation into TDIA through a cross-layer design, aiming to further improve performance.

### 4.1. TDIA-Based Transmission Scheduling

With the traditional TDIA, the optimal transmission schedule to prevent collision on a useful signal is not unique. Hence, for the UWA networks considered in this study, the optimal schedule is considered as the one that minimizes the total transmission time, obtained by solving

(17a) $${\left\{T_{m}^{\left(l\right)}\right\}}_{1\leq m\leq N,1\leq l\leq L}=\arg\min T_{\mathrm{total}}$$

(17b) $$\begin{array}{cc} s.t.\; & |\Delta_{mkm}^{\left(l_{m},l_{k}\right)}|\geq T_{\mathrm{bl}},k\in I\left(m\right); \end{array}$$

(17c) $$\begin{array}{cc} \; & T_{m}^{\left(l_{2}\right)}-T_{m}^{\left(l_{1}\right)}\geq T_{\mathrm{bl}},l_{1}<l_{2}; \end{array}$$

where constraints (17b) and (17c) ensure that useful signals remain collision-free and that each transmitter sends only one block at a time, respectively. In addition, by minimizing the objective (17a), the interference signals will be squeezed together as much as possible since there is no restriction on ToA difference between any two interference blocks. Once the optimal schedule is derived, all transmitters operate at their maximum power levels, which could maximally improve the network throughput.

According to [33], an MILP problem can be constructed by reformulating the optimization problem (17) with transformation techniques. Specifically, according to (13) and (14), the optimization objective can be reformulated from

(18) $$\arg\min\max_{i,j,m,l_{i},l_{j}}\left\{t_{im}^{\left(l_{i}\right)}+T_{\mathrm{bl}}-t_{jm}^{\left(l_{j}\right)}\right\}$$

to

(19a) $$\begin{array}{cc} \; & \arg\min\rho \end{array}$$

(19b) $$\begin{array}{cc} s.t.\; & t_{im}^{\left(l_{i}\right)}+T_{\mathrm{bl}}-t_{jm}^{\left(l_{j}\right)}\leq \rho ; \end{array}$$

where ρ is an auxiliary variable.

For the constraint (17b), according to (1), (2), and (4), the ToA $\Delta_{mkn}^{\left(l_{m},l_{k}\right)}$ can be expressed as a linear transformation of the transmission starting time within the transmission schedule. According to [33], the constraint (17b) can be converted to

(20a) $$\begin{array}{cc} \Delta_{mkn}^{\left(l_{m},l_{k}\right)}+\Gamma_{mk}^{\left(l_{m},l_{k}\right)}\alpha_{mk}^{\left(l_{m},l_{k}\right)} & >T_{\mathrm{bl}}; \end{array}$$

(20b) $$\begin{array}{cc} -\Delta_{mkn}^{\left(l_{m},l_{k}\right)}+\Gamma_{mk}^{\left(l_{m},l_{k}\right)}\left(1-\alpha_{mk}^{\left(l_{m},l_{k}\right)}\right) & >T_{\mathrm{bl}}; \end{array}$$

(20c) $$\begin{array}{c} \alpha_{mk}^{\left(l_{m},l_{k}\right)}\in \left\{0,1\right\} \end{array}$$

where $\alpha_{mk}^{\left(l_{m},l_{k}\right)}$ is a binary variable and $\Gamma_{mk}^{\left(l_{m},l_{k}\right)}$ is a relatively large constant.

Hence, by further introducing auxiliary variables to the constraint (17b) and (17c), the MILP formulation of the TDIA can be established as

(21a) $$\begin{array}{cc} \; & \arg\min\rho \end{array}$$

(21b) $$\begin{array}{cc} s.t.\; & \Delta_{mkn}^{\left(l_{m},l_{k}\right)}+\Gamma_{mk}^{\left(l_{m},l_{k}\right)}\alpha_{mk}^{\left(l_{m},l_{k}\right)}>T_{\mathrm{bl}}; \end{array}$$

(21c) $$\begin{array}{cc} \; & -\Delta_{mkn}^{\left(l_{m},l_{k}\right)}+\Gamma_{mk}^{\left(l_{m},l_{k}\right)}\left(1-\alpha_{mk}^{\left(l_{m},l_{k}\right)}\right)>T_{\mathrm{bl}}; \end{array}$$

(21d) $$\begin{array}{cc} \; & \alpha_{mk}^{\left(l_{m},l_{k}\right)}\in \left\{0,1\right\} \end{array}$$

(21e) $$\begin{array}{cc} \; & T_{m}^{\left(l_{2}\right)}-T_{m}^{\left(l_{1}\right)}\geq T_{\mathrm{bl}},l_{1}<l_{2}; \end{array}$$

(21f) $$\begin{array}{cc} \; & t_{im}^{\left(l_{i}\right)}+T_{\mathrm{bl}}-t_{jm}^{\left(l_{j}\right)}-\rho \leq 0. \end{array}$$

where the optimization variables are $\left\{T_{m}^{\left(l\right)}\right\}$, $\left\{\alpha_{mk}^{\left(l_{m},l_{k}\right)}\right\}$, and ρ. As a result, the objective function and constraints in (21) are linear with respect to the transmission starting time $\left\{T_{m}^{\left(l\right)}\right\}$. Thus, the optimal transmission schedule can be efficiently and accurately determined by addressing (21) using the mature MILP solvers [34]. In the next section, we introduce the optimization problem under QIA, which is then solved by leveraging the solver foundation established for TDIA.

### 4.2. QIA with Cross-Layer Design

**Figure 5:**
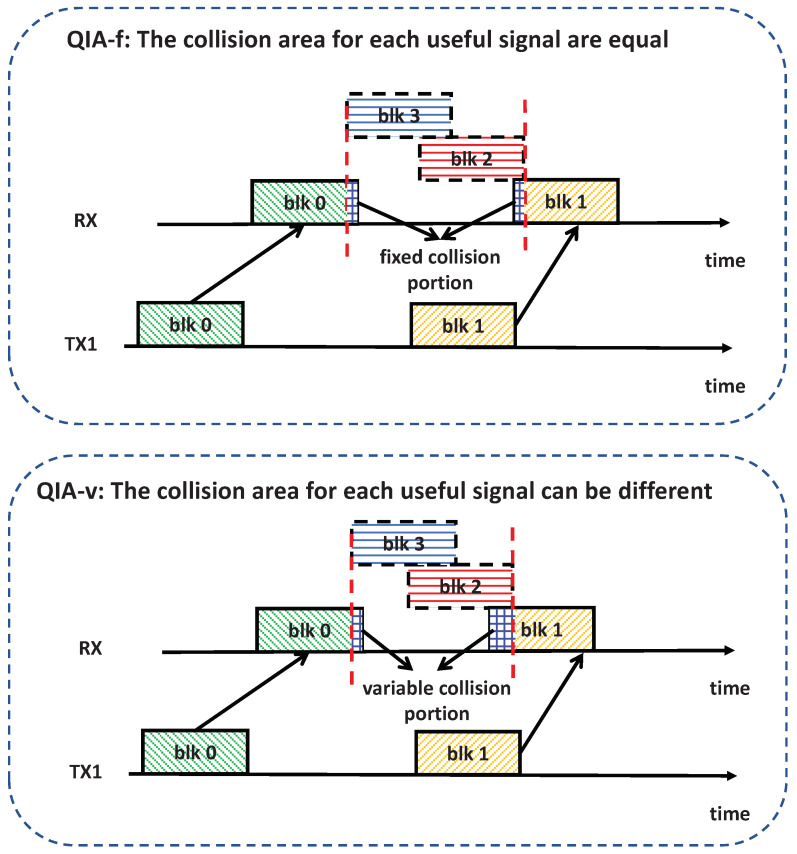
An illustration of QIA-f and QIA-v.

As illustrated in Figure 5, the proposed QIA mechanism permits useful signals to be partially contaminated by interference signals, but only within an acceptable threshold. It indicates that the absolute value of the ToA difference $|\Delta_{mkm}^{\left(l_{m},l_{k}\right)}|$ can be smaller than the duration of the signal block. In this work, we propose two algorithms, i.e., QIA with a fixed allowable collision level (QIA-f) and QIA with a variable allowable collision level (QIA-v). The two algorithms differ in how the maximal collision level of useful signals is managed, as depicted in Figure 5. In QIA-f, the maximal collision level for a useful signal remains fixed, whereas in QIA-v, this level is variable and subject to optimization, providing greater flexibility for throughput enhancement. The detailed descriptions of the two proposed algorithms are presented in the following.

#### 4.2.1. QIA-f

In this scenario, we assume that all the useful signal blocks can potentially be affected by interference from other signals, limited to a fixed level of collision in the time domain. Additionally, we assume that the maximal allowed collision levels for all the useful signal blocks are the same, indicating that they could be exposed to an equal degree of interference in the time domain. Denote η as the collision factor, which is a pre-defined parameter that controls the time-domain collision level for all the useful signal blocks. Accordingly, the constraint (17b) on the ToA differences between received useful and interference signals at receiver *m* is adjusted to

(22) $$|\Delta_{mkm}^{\left(l_{m},l_{k}\right)}|\geq \left(1-\eta \right)T_{\mathrm{bl}},k\in \left(m\right).$$

Here, the collision factor η(0≤η≤1) governs the collision intensity between useful and interference signals. A larger value of η indicates a higher allowable collision intensity, while η=0 corresponds to the traditional TDIA approach, which strictly prohibits any collision.

The optimization problem for QIA-f with power allocation incorporated can be established as

(23a) $$\begin{array}{cc} \{T_{m}^{\left(l\right)}, & P_{m}^{\left(l\right)}{\}}_{1\leq m\leq N,1\leq l\leq L}=\arg\max C_{\mathrm{sum}} \end{array}$$

(23b) $$\begin{array}{cc} s.t.\; & P_{\min}\leq P_{\mathrm{tx},m}^{\left(l\right)}\leq P_{\max}; \end{array}$$

(23c) $$\begin{array}{cc} \; & |\Delta_{mkm}^{\left(l_{m},l_{k}\right)}|\geq \left(1-\eta \right)T_{\mathrm{bl}},k\in I\left(m\right); \end{array}$$

(23d) $$\begin{array}{cc} \; & T_{m}^{\left(l_{2}\right)}-T_{m}^{\left(l_{1}\right)}\geq T_{\mathrm{bl}},l_{1}<l_{2}; \end{array}$$

(23e) $$\begin{array}{cc} \; & P_{\mathrm{rx},mm}^{\left(l\right)}\geq P_{\mathrm{th}}. \end{array}$$

Compared to the optimization problem (16), the feasible region in (23) for the transmission starting time is heavily reduced due to the fixed allowable collision level. However, the complexity of its objective function, which involves nonlinear calculations like the SINR in (6), presents a challenge for direct resolution.

To make the optimization problem (23) more manageable, the transmission starting times are first coarsely determined to minimize the total reception time as

(24a) $${\left\{T_{m}^{\left(l\right)}\right\}}_{1\leq m\leq N,1\leq l\leq L}=\arg\min T_{\mathrm{total}}$$

(24b) $$\begin{array}{cc} s.t.\; & |\Delta_{mkm}^{\left(l_{m},l_{k}\right)}|\geq \left(1-\eta \right)T_{\mathrm{bl}}; \end{array}$$

(24c) $$\begin{array}{cc} \; & T_{m}^{\left(l_{2}\right)}-T_{m}^{\left(l_{1}\right)}\geq T_{\mathrm{bl}}. \end{array}$$

Similarly to (17), the transmission schedule, denoted as $T_{m,\mathrm{opt}}^{\left(l\right)}$, can be efficiently determined by solving a reformulated MILP problem.

The obtained transmission schedule $T_{m,\mathrm{opt}}^{\left(l\right)}$ is then further refined with transmission power to alleviate the impact of minor collisions associated with QIA. This refinement is achieved by solving the following optimization problem:

(25a) $$\begin{array}{cc} \{T_{m}^{\left(l\right)}, & P_{m}^{\left(l\right)}{\}}_{1\leq m\leq N,1\leq l\leq L}=\arg\max C_{\mathrm{sum}} \end{array}$$

(25b) $$\begin{array}{cc} s.t.\; & P_{\min}\leq P_{\mathrm{tx},m}^{\left(l\right)}\leq P_{\max}; \end{array}$$

(25c) $$\begin{array}{cc} \; & |T_{m}^{\left(l\right)}-T_{m,\mathrm{opt}}^{\left(l\right)}|\leq \epsilon ; \end{array}$$

(25d) $$\begin{array}{cc} \; & |\Delta_{mkm}^{\left(l_{m},l_{k}\right)}|\geq \left(1-\eta \right)T_{\mathrm{bl}}; \end{array}$$

(25e) $$\begin{array}{cc} \; & P_{\mathrm{rx},mm}^{\left(l\right)}\geq P_{\mathrm{th}}; \end{array}$$

where the constraint (25c) enforces that the final optimal transmission starting time, $T_{m}^{\left(l\right)}$, must remain within an ϵ-neighborhood of the preliminary schedule $T_{m,\mathrm{opt}}^{\left(l\right)}$. Hence, the feasible region of the problem (25) is significantly narrowed down compared to the broader formulation in (16), allowing for faster convergence. Leveraging this reduced search space, the problem can be effectively solved using the stochastic gradient decent (SGD) method, as outlined in [35].

The proposed QIA-f approach approximates a solution to the problem (16) by addressing two sub-problems sequentially. Initially, a TDIA-based problem is solved to determine a preliminary transmission schedule. This step focuses on minimizing the total reception duration while maximizing interference signal overlap, thereby achieving higher network throughput from an MAC layer perspective. Subsequently, a joint refinement process is carried out to optimize both the transmission schedule and power within a more constrained feasible region, characterized by the preliminary schedule obtained in the first step. This refinement aims to mitigate the adverse effects of interference caused by minor collisions of useful signals. The process resembles a regret mechanism, compensating for the limitations of the initial greedy scheduling approach. Leveraging the cross-layer design, the two-step approach takes the advantage of a significantly reduced feasible region, with the ultimate goal of maximizing transmission capacity.

#### 4.2.2. QIA-v

The proposed QIA-f algorithm aims to restrict the interference level on useful signals by imposing a fixed maximal allowable collision factor. However, this approach may result in missing the optimal collision pattern in the time domain since each transmitted signal block could experience the unique transmission loss based on the location of the transmitter. Therefore, we further investigated how to introduce more collision patterns for useful signals by considering variable maximal allowable collision levels.

As the received power of each signal block can be adjusted, we considered assigning a variable maximal allowable collision level for each pair of useful and interference signal blocks. It allows for more flexibility in exploring the collision patterns and for a better adaptation to the specific transmission loss of each signal block. The constraint (17b) in this scenario can be redefined as

(26) $$|\Delta_{mkm}^{\left(l_{m},l_{k}\right)}|\geq \left(1-\eta_{mkm}^{\left(l_{m},l_{k}\right)}\right)T_{\mathrm{bl}}$$

where the collision factor $\eta_{mkm}^{\left(l_{m},l_{k}\right)}$ is exploited to determine the collision pattern between the $l_{m}$th and $l_{k}$th signal blocks from the *m*th and *k*th transmitters, respectively, both of which are received by the *m*th receiver. The variability of the collision factor indicates the adaptive design of the interference level in the time domain for each colliding instance. The proposed QIA-v algorithm intended to jointly determine the transmission power and schedule can be cast as

(27a) $$\begin{array}{cc} \{T_{m}^{\left(l\right)}, & P_{m}^{\left(l\right)}{\}}_{1\leq m\leq N,1\leq l\leq L}=\arg\max C_{\mathrm{sum}} \end{array}$$

(27b) $$\begin{array}{cc} s.t.\; & P_{\min}\leq P_{\mathrm{tx},m}^{\left(l\right)}\leq P_{\max}; \end{array}$$

(27c) $$\begin{array}{cc} \; & |\Delta_{mkm}^{\left(l_{m},l_{k}\right)}|\geq \left(1-\eta_{mkm}^{\left(l_{m},l_{k}\right)}\right)T_{\mathrm{bl}}; \end{array}$$

(27d) $$\begin{array}{cc} \; & T_{m}^{\left(l_{2}\right)}-T_{m}^{\left(l_{1}\right)}\geq T_{\mathrm{bl}},l_{1}<l_{2}; \end{array}$$

(27e) $$\begin{array}{cc} \; & P_{\mathrm{rx},mm}^{\left(l\right)}\geq P_{\mathrm{th}}. \end{array}$$

The main distinction between the optimization problems (23) and (27) lies in the constraints (27c) and (23c), where variable collision factors are considered. If those variable factors $\eta_{mkm}^{\left(l_{m},l_{k}\right)}$ are determined, the optimization problem (27) can be solved straightforwardly in a similar way to the QIA-f algorithm, where the two-step optimization strategy can be applied. However, how to choose appropriate collision factors becomes a crucial problem, as they significantly influence the collision patterns and the overall system performance. Properly tuning the collision factors can lead to optimized transmission power and schedule, which could help improve the network throughput and efficiency.

From the machine learning perspective, these collision factors can be treated as hyperparameters, and selecting those factors can be regarded as hyperparameter tuning [36]. Denote *F* as the procedure for solving the optimization problem (27), which returns the optimal transmission capacity. The input–output relation of *F* can be mathematically described as

(28) $$C_{\mathrm{opt}}=F\left({\left\{\eta_{mkm}^{\left(l_{m},l_{k}\right)}\right\}}_{1\leq m\leq N,k\in I\left(m\right),1\leq l_{m},l_{k}\leq L}\right).$$

**Figure 6:**
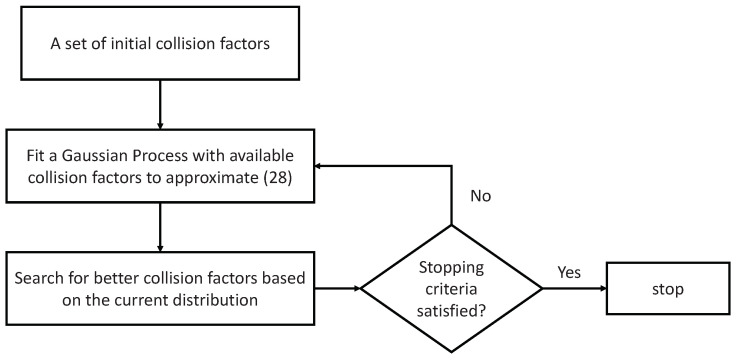
An illustration of BO to seek for collision factors.

It indicates that, given a set of collision factors $\eta_{mkm}^{\left(l_{m},l_{k}\right)}$, one can obtain an optimized value of transmission capacity and the optimal transmission power and schedule under the corresponding hyperparameters. Thus, the relationship between performance (i.e., transmission capacity) and hyperparameters (i.e., collision factors) is established. Rather than directly searching for the optimal collision factors, a process characterized by low efficiency and high computational complexity, we treat the selection of appropriate collision factors as a hyperparameter tuning problem, a concept extensively studied in the machine learning research community. In this work, we propose leveraging Bayesian optimization (BO) [37] to efficiently determine the optimal collision factors. The core concept involves utilizing a Gaussian process to model the distribution of the resulting throughput across the collision factors, based on a limited number of samples. Specifically, in each search iteration of BO, as the current collision factors are considered as known, we can exploit the proposed QIA-f approach with these collision factors to solve for transmission schedule and transmission power. The implementation of BO for tuning the collision factors is briefly summarized in Figure 6. For further information about hyperparameter tuning and BO, please refer to [38,39] and references therein.

**Figure 7:**
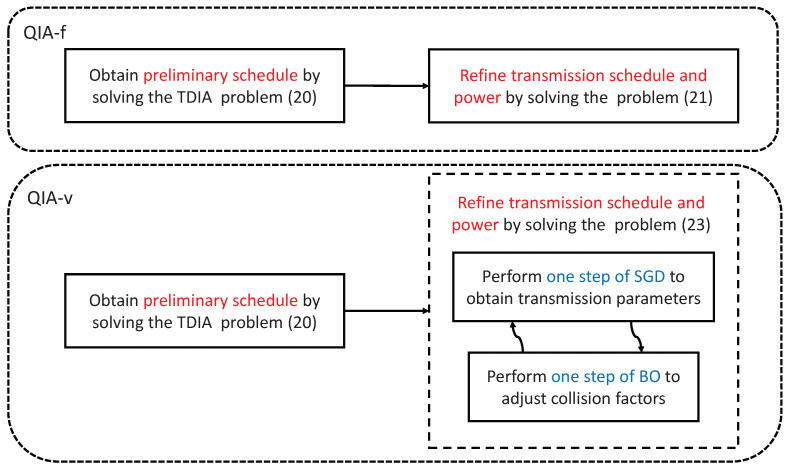
An illustration of workflows for QIA-f and QIA-v.

The workflows of QIA-f and QIA-v are depicted in Figure 7, highlighting their distinctions in the refinement process for both transmission schedule and power allocation. In QIA-f, the refinement is straightforward: the transmission schedule and power levels are derived solely by solving the optimization problem (25) using the preliminary transmission schedule as input. This step employs the SGD method to optimize both variables simultaneously.

In contrast, QIA-v employs an iterative refinement process. Each iteration involves two steps:

1. SGD Step: Solve the optimization problem (27) to optimize the transmission schedule and power based on the current set of collision factors.
2. BO Step: Treat the collision factors as optimization variables and employ BO to solve the optimization problem (28), updating the collision factors.

These two steps are repeated iteratively until the improvement in throughput becomes marginal. This iterative mechanism enables QIA-v to dynamically adapt the collision factors while optimizing the schedule and power, resulting in potential performance improvement compared to QIA-f.

## 5. Performance Evaluation

The performance of the proposed QIA-based methods is evaluated and compared through simulations. In the simulation setup, UWA network nodes are randomly and uniformly distributed within a radius of 4 kilometers. The algorithms are tested on networks consisting of multiple transmitter–receiver pairs, which are randomly assigned for each configuration. The throughput performance is determined by averaging results over 1000 random network layouts. A summary of the common simulation parameters is provided in Table 1.

In addition to the baseline TDIA method, a global optimization solver, namely the genetic algorithm (GA), is used as a competing method to directly solve the optimization problem (16). For the proposed QIA-f, the collision factor is fixed at η=0.2, whereas the proposed QIA-v utilizes the BO algorithm to optimize the collision factors concurrently.

We further define the averaged transmission signal-to-noise ratio (SNR) for the transmitters as

(29) $$\mathrm{TSNR}=\frac{1}{NL}\sum_{l=1}^{L}\sum_{m=1}^{N}\frac{P_{\mathrm{tx},m}^{\left(l\right)}}{P_{0}}$$

### 5.1. Performance Comparison of Transmission Capacity

**Figure 8:**
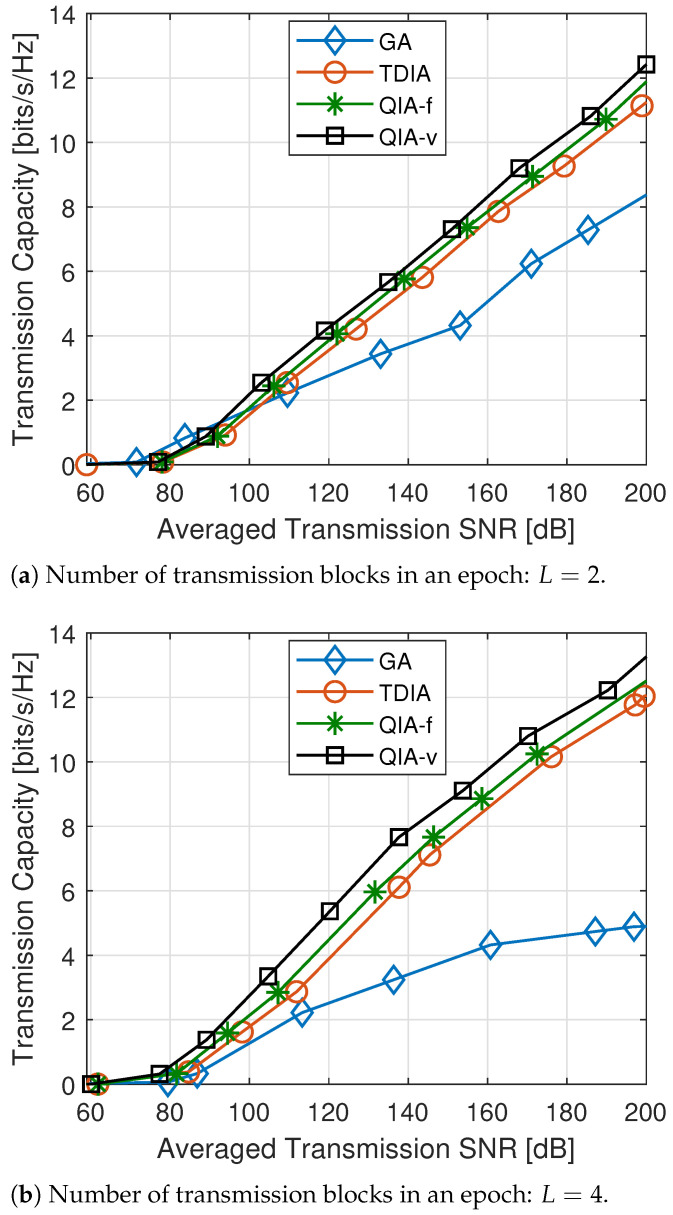
Throughput comparison under different methods.

The comparisons of network throughput, i.e., averaged transmission capacity in bits/s/Hz, with L=2 and L=4 by using different methods, are depicted in Figure 8. The simulation results demonstrate that the proposed QIA-f and QIA-v methods consistently outperform the baseline methods as the transmission SNR increases. Specifically, for L=2 with two transmission blocks per link, the performance gains—defined as the reduction in transmission SNR required to achieve the same throughput—are approximately 3 dB and 6 dB for QIA-f and QIA-v, respectively, compared to the baseline TDIA. Similarly, for L=4, QIA-f and QIA-v achieve gains of around 6 dB and 10 dB, respectively, over TDIA. These findings highlight that as the number of transmitted signal blocks increases, the proposed methods become increasingly effective. The additional signal blocks introduce more collision patterns, creating more opportunities for feasible idle periods to support concurrent transmissions. Furthermore, QIA-v achieves higher throughput than QIA-f by incorporating variable collision factors for each transmitted signal block, providing greater degrees of freedom to optimize network throughput. These results validate the efficacy of integrating cross-layer design principles into TDIA to enhance network performance.

Regarding the GA method, it demonstrates superior performance compared to other approaches at relatively low transmission power levels, though the overall throughput magnitude remains low. In scenarios with limited transmission power, weaker interference signals enable the global optimization capability of GA to align interference more effectively with useful signals. On the other hand, TDIA-based methods prioritize scheduling to align received interference, even when it is relatively weak. This strategy, while effective in managing interference, limits the time available for transmitting useful signals, thereby resulting in reduced transmission capacity. However, as the transmission power increases, QIA-based methods become advantageous. Their ability to incorporate slight collisions in the scheduling process, combined with power allocation at the physical layer, allows for a more efficient utilization of resources and results in enhanced network throughput.

### 5.2. Comparison of Computation Complexity and Convergence

The computational complexity, in CPU processing time (seconds), is summarized in Table 2. The results are obtained using a computer with a 1.90-GHz i7-8650U CPU (Intel, Santa Clara, CA, USA) and 8 GB of RAM. It indicates that TDIA consumes the least CPU time compared to QIA-f and QIA-v, attributed to its efficient MILP solution. Integrating MILP with an additional step of nonlinear optimization, QIA-f consumes more time than TDIA. However, as the transmission scheduling time obtained in the first step is already suboptimal, this accelerates the joint optimization of transmission slots and power in the second step, resulting in only a marginal increase in time compared to TDIA. By further incorporating the BO process, QIA-v requires additional time to optimize the collision factor, yielding further gains in optimal throughput. Nonetheless, the relatively low computation time of the proposed approaches demonstrates their feasibility for practical implementation.

**Figure 9:**
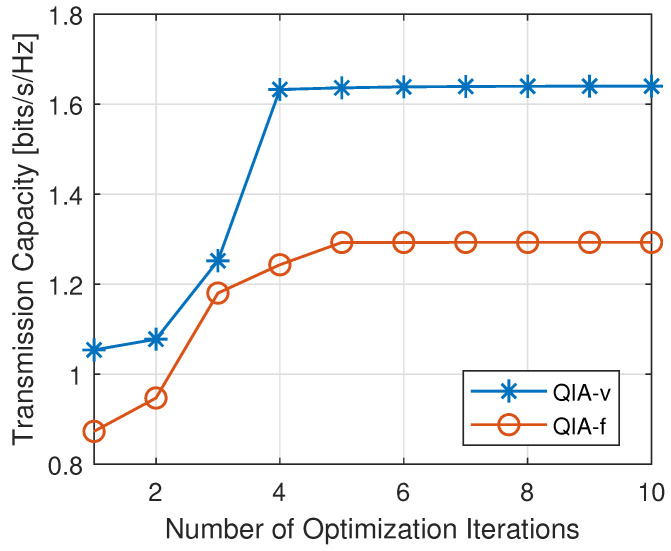
An illustration of capacity convergence during the refinement step.

Figure 9 illustrates the convergence behavior of the capacity achieved by QIA-f and QIA-v during the refinement process, showcasing how capacity improves across optimization iterations. Both proposed approaches exhibit rapid convergence, typically within fewer than five iterations. Notably, QIA-v outperforms QIA-f in both convergence speed and final capacity. This improvement can be attributed to its integration of BO, which dynamically searches for appropriate collision factors during the second step. This adaptive mechanism allows QIA-v to more effectively align the optimization process with the interference structure, thereby improving throughput and overall efficiency.

It would be beneficial to include additional details and clarification on the content and significance of Figure 8.

### 5.3. QIA-f with Different Collision Factors

**Figure 10:**
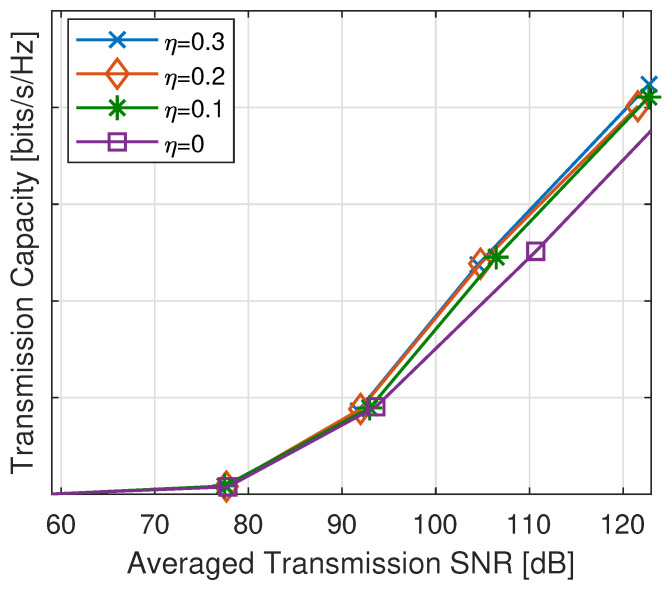
Throughput comparison of QIA-f with different collision factors η.

We further analyzed the performance of the proposed QIA-f algorithm under varying collision factors, i.e., η∈{0,0.1,0.2,0.3}, to study the impact of collision intensity on QIA-f and to justify the advantages of QIA-v. Note that η=0 corresponds to the traditional TDIA case. As illustrated in Figure 10, increasing the collision factor η enhances the transmission capacity; however, the rate of improvement diminishes at higher levels of permissible collision intensity. This behavior can be attributed to two key factors. First, as collisions intensify, the capacity of power allocation to effectively mitigate interference diminishes, limiting its ability to offset the adverse effects of increased collisions. As a result, even with a higher permissible collision intensity, the QIA-f schedule tends to induce lower actual collision levels on useful signals, leading to marginal performance gains. Second, a larger collision factor η expands the feasible region for QIA-f, making the optimization process more complex. In practice, determining an optimal collision factor often involves trial and error, which can be both time-consuming and inefficient. In such scenarios, QIA-v demonstrates a clear advantage by dynamically adjusting the feasible region through adaptive expansion or contraction. This adaptability enables QIA-v to align more effectively with the specific interference structure of each transmission link, thereby enhancing performance in challenging network conditions without requiring a predetermined collision factor.

### 5.4. QIA-v with Different Searching Strategies for Collision Factors

**Figure 11:**
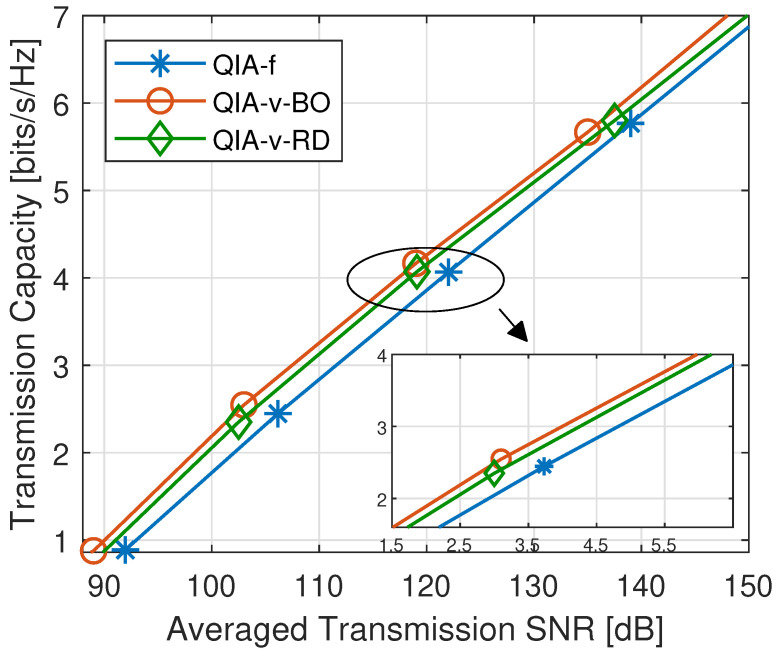
Throughput comparison with QIA-v-BO and QIA-v-RD.

Compared to QIA-f, the search strategy for appropriate collision factors in QIA-v plays a crucial role in the throughput enhancement. We then compare the performance of QIA-v with two popular searching mechanisms for hyperparameter tuning, i.e., QIA-v with BO (QIA-v-BO) and QIA-v with random search [38] (QIA-v-RD). The throughput comparison of these methods is presented in Figure 11. It can be observed that QIA-v-BO outperforms QIA-v-RD slightly, achieving the performance gain of approximately 1 dB. The exploitation versus exploration mechanism employed by BO enables a more organized and systematic search for the optimal collision factors, making the searching process more effective compared to the purely randomized sampling-based approach of QIA-v-RD.

### 5.5. Transmission d.o.f. Under Different System Setups

We then studied the performance of the proposed QIA-v algorithm in terms of interference suppression under different system setups, which involves different numbers of blocks to be transmitted by a transmitter and different numbers of active links in the network. To assess the capability of the proposed method in handling interference, we define the transmission d.o.f. as the ratio of the total transmission capacity to the capacity with the averaged transmission SNR, i.e.,

(30) $$\rho =\frac{C_{\mathrm{sum}}}{\frac{1}{2}\log_{2}\left(1+\mathrm{TSNR}\right)}.$$

This metric characterizes the freedom to reduce the impact of interference on the overall transmission capacity when utilizing the proposed power allocation and scheduling method.

**Figure 12:**
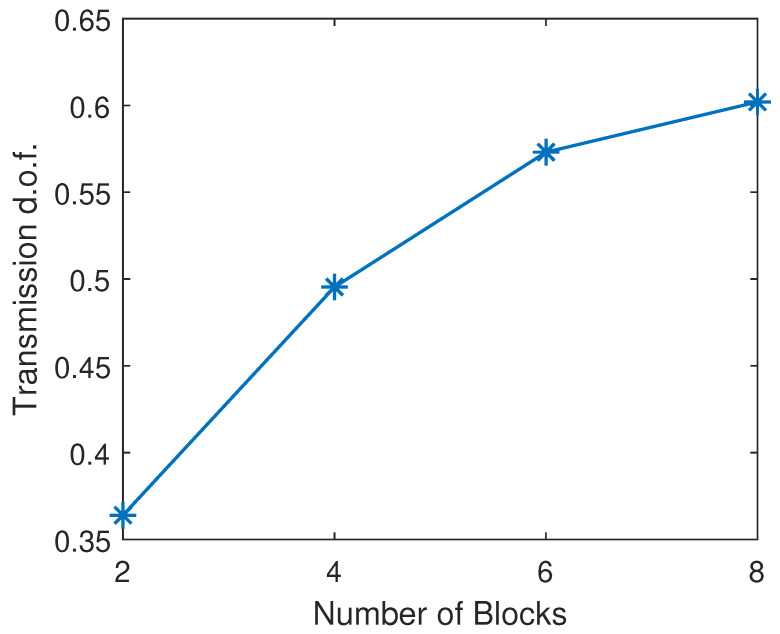
Transmission d.o.f. by QIA-v with different numbers of transmitted blocks.

The transmission d.o.f. with different numbers of transmitted signal blocks is shown in Figure 12, where the number of active links is set to 4. One can see that the transmission d.o.f. grows monotonically with the number of blocks. It indicates that, by allowing more signal blocks to be transmitted in a round, the proposed method could promise a greater capability to mitigate the interference. However, it is worth noting that the improvement in the d.o.f. becomes less significant when further increasing the number of blocks in a transmission round.

**Figure 13:**
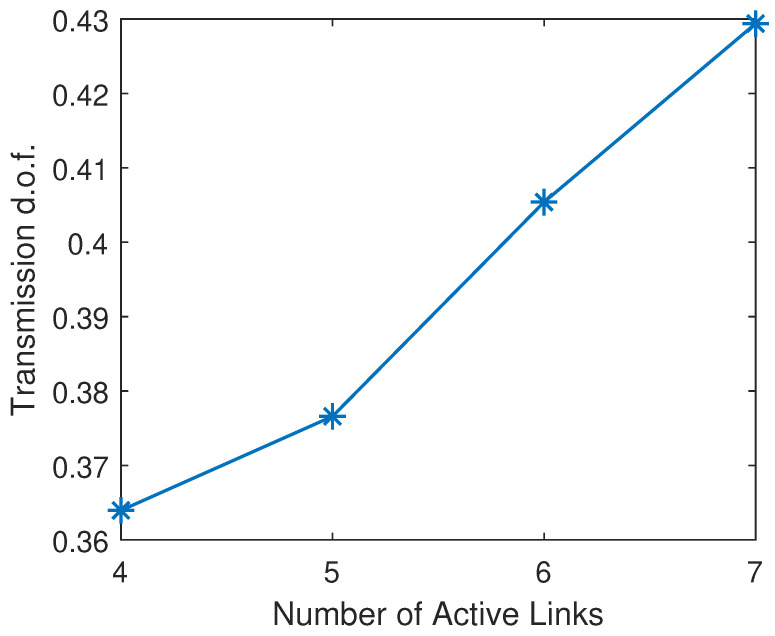
Transmission d.o.f. by QIA-v with different numbers of active links.

Similarly, the transmission d.o.f. also increases with the number of active links, as depicted in Figure 13. However, compared to Figure 12, the improvement in d.o.f. achieved by a greater number of active links is insignificant, where the absolute increment in transmission d.o.f. is minor. This is due to the fact that, as the number of active links increases, more interference sources should be considered while the capability of the proposed method in compromising interfering links becomes weaker, compared to the case with a greater number of transmitted blocks.

**Figure 14:**
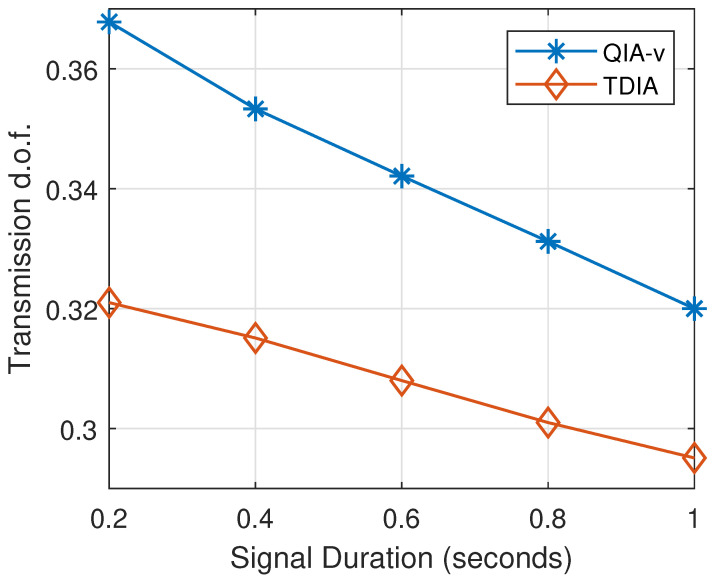
Transmission d.o.f. with different values of signal duration.

In practice, the signal duration varies across different UWANs and typically ranges on the order of hundreds of milliseconds [32]. The transmission d.o.f. of the proposed QIA-v compared to TDIA are presented in Figure 14, where the numbers of active links and signal blocks are set to 4. The results demonstrate that the performance of both QIA-v and TDIA degrades as the signal duration increases. This is because longer signal durations, coupled with fixed transmission latencies, reduce the available time for concurrent transmissions with effective interference alignment, thereby decreasing the d.o.f. for throughput optimization. Nonetheless, within practical signal duration ranges, the proposed QIA-v consistently outperforms TDIA. However, as the signal duration further increases, the performance gain of QIA-v diminishes relative to TDIA. This is attributed to the reduced likelihood of interference alignment, which limits opportunities for throughput improvement through joint transmission scheduling and power allocation.

## 6. Discussions

By strategically leveraging the large propagation delays associated with acoustic transmissions in underwater environments, the TDIA mechanism emerges as a classic methodology for enhancing throughput in UWA networks from the perspective of the MAC layer [14,16,21], where the collisions of transmissions are strictly prohibited. The core idea is to adjust the transmission time such that interference signals arriving at the receiver can be aligned, thereby creating more free time slots for useful signals [40]. Since the propagation delays between the nodes can be any value, many existing works either consider adjusting the duration of transmission signals based on the spatial layout of the network nodes [14,40] or simply assume that the delays are multiples of integers of the duration of the time slot [16]. These strategies aim to achieve the perfect alignment of interfered time slots in which the interference signals are received. However, they may not be applicable in practical systems due to considerations such as the fixed packet duration in UWA modems [41] or the assumption of propagation delays being integers of time slots not holding, potentially resulting in signal collisions [17]. The approach proposed in [22] can be effective in networks with arbitrary propagation delays while a vacuum time duration similar to the scenario depicted in Figure 2 persists, leading to time wastage during transmissions. Hence, in this study, a transmission scheduling strategy is considered to further enhance network throughput and reduce the vacuum time duration while accounting for arbitrary propagation delays.

To further reduce the vacuum time duration, it is anticipated that signal collisions will inevitably occur, necessitating the relaxation of stringent constraints on collision-free signal reception from the perspective of the MAC layer. To mitigate the impact of collisions, the proposed QIA method integrates power allocation in TDIA to minimize the throughput degradation induced by collisions. The performance evaluation demonstrates that introducing minor collisions through scheduling, combined with power allocation to compensate for these collisions, is a viable strategy for enhancing throughput performance. In comparison to another state-of-the-art TDIA-based method adapted for single-hop networks [22], a significant performance gain of approximately 6 dB is observed. These results suggest that the proposed QIA method can allocate more temporal resources for useful signals while simultaneously mitigating signal collisions through power allocation, thereby achieving higher throughput performance compared to the sole use of TDIA within the MAC layer framework. Fundamentally, the performance gain can be attributed to the cross-layer design [42], where parameters across different network layers are jointly optimized.

In addition, the allowable collision level is a pivotal parameter for collision control and mitigation. Relatively large collision factors could lead to greater time savings but also introduce more interference regions in time. Selecting the appropriate collision factors can be challenging since they should be determined according to the colliding pattern, which is influenced by the spatial distribution of the network nodes [43]. In this study, rather than relying on predetermined collision factors, a more structured approach based on a statistically directed optimization strategy is designed for searching optimal allowable collision levels during the adjustment to power and transmission schedule. Integrating Bayesian optimization to seek the optimal collision factors has been proven to be more effective than simply utilizing fixed factors or employing the naive random search for these factors, which coincides with the existing knowledge in the machine learning research community [38]. As many existing works for UWA networks consider predetermined parameters [44,45], this study suggests that greater improvement can be achieved by optimally adjusting these parameters.

Recently, more works [27,46,47] consider reinforcement learning (RL) for MAC design in UWA networks, where an agent learns the optimal transmission schedule based on the interaction with the environment through online learning, leading to superior performance. However, to the best of our knowledge, its application for the cross-layer design in UWA networks is rarely investigated. This study potentially provides the basic system model to be integrated into the RL framework. In the future, we will investigate how to exploit RL for the QIA problem to further enhance throughput performance.

## 7. Conclusions and Future Works

This work investigated a TDIA-based mechanism enhanced with a cross-layer design to enhance throughput in UWA networks by considering arbitrary propagation delays. Building on traditional TDIA, which focuses particularly on transmission scheduling at the MAC layer, the proposed approach allows for a relaxed transmission schedule that permits minor interference with useful signals. To address the challenges posed by the large feasible region induced by the cross-layer design, a two-step optimization strategy to jointly refine the transmission schedule and optimize transmission power was proposed. In addition, two algorithms, i.e., QIA-f and QIA-v, were proposed, which differed in how to select maximal allowable collision levels for better collision patterns. The cross-layer design, along with the consideration of introducing better collision patterns, achieved decent performance gains over the competing methods. The effectiveness of the proposed algorithms in mitigating interference was validated by their ability to achieve a higher transmission throughput.

In this study, large-scale channel attenuation is considered. However, underwater acoustic channels exhibit complex dynamics due to node mobility and environmental variations. Future work could focus on addressing more complex and time-varying channel conditions, building on the framework of QIA. Additionally, advanced interference management techniques, such as beamforming and network coding, could be integrated with the proposed QIA to provide additional degrees of freedom for further enhancing network throughput.

## Figures and Tables

**Table 1:** Simulation parameters for performance evaluation.

Parameters	Values
Signal duration	0.2 s
Transmission center frequency	12 kHz
Transmission bandwidth	10 kHz
Sound speed in water	1500 m/s

**Table 2:** Comparison of computation time in seconds.

	L=2	L=4	L=6
GA	5.6	13.6	37.1
TDIA	0.12	0.27	0.51
QIA-f	0.17	0.31	0.63
QIA-v	0.25	0.37	0.71

## Data Availability

Data are contained within the article.
